# Phytotherapeutic Information on Plants Used for the Treatment of Tuberculosis in Eastern Cape Province, South Africa

**DOI:** 10.1155/2014/735423

**Published:** 2014-04-22

**Authors:** I. O. Lawal, D. S. Grierson, A. J. Afolayan

**Affiliations:** Medicinal Plant and Economic Development (MPED) Research Centre, Department of Botany, University of Fort Hare, Private Bag X1314, Alice 5700, South Africa

## Abstract

The current rate of deforestation in Africa constitutes a serious danger to the future of medicinal plants on this continent. Conservation of these medicinal plants in the field and the scientific documentation of our knowledge about them are therefore crucial. An ethnobotanical survey of plants used for the treatment of tuberculosis (TB) was carried out in selected areas of the Eastern Cape, South Africa. These areas were Hala, Ncera, Sheshegu, and Gquamashe, all within the Nkonkobe Municipality. One hundred informants were interviewed. The survey included the identification of scientific and vernacular names of the plants used for treatment of TB as well as the methods of preparation and administration, the part used, dosage, and duration of treatment. The survey revealed 30 plants belonging to 21 families which are commonly used by traditional healers for the treatment of TB and associated diseases. Of these plants *Clausena anisata, Haemanthus albiflos,* and *Artemisia afra* were the most cited. The leaves were the most common part used in the medicinal preparations. Our findings are discussed in relation to the importance of the documentation of medicinal plants.

## 1. Introduction

Tuberculosis (TB) is a fearful disease in developing nations, especially in the Asian and African continents, probably due to their inadequate means for the management and treatment of the disease. It is caused by a bacterium called* Mycobacterium tuberculosis.* TB infects nine million people every year most of them being children, and it leads to approximately two million deaths annually [1, 2]. These statistics are likely to increase in future because the human immunodeficiency virus (HIV) is entwined with TB and also due to the surfacing of multidrug-resistant strains of TB organisms [3–5].

TB is a very common disease in South Africa. According to reports, it is the fifth largest cause of death in this country [6, 7]. For example, approximately 285,000 cases of TB were reported in South Africa in 2005. In fact, South Africa has the seventh highest number of people suffering from TB in the world and the second highest in Africa [7]. In addition, the country has the fifth highest burden of drug-resistant tuberculosis cases in the world [8].

Africa is endowed with an enormous wealth of plant resources [9]. Herbal remedies from these plants have contributed to the reduction of excessive mortality, morbidity, and disability brought about by diseases such as HIV/AIDS, malaria, tuberculosis, sickle cell anaemia, diabetes, mental, disorder and microbial infections [10]. In addition to treating infectious diseases, phytomedicines have been reported to limit the side effects associated with synthetic antimicrobial drugs [11].

The Eastern Cape Province of South Africa is known for its richness in plant species [13]. The inhabitants of this province have a long history of traditional plant usage for the treatment of various diseases including TB [14]. Herbal medicine, being a significant element in the cultural patrimony, still remains the main recourse for a large majority of people for addressing health problems.

**Figure 1:**
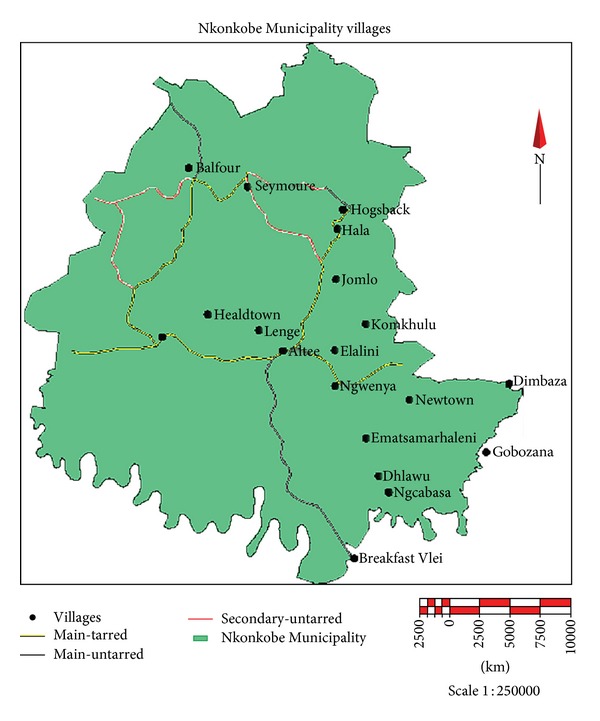
Nkonkobe Municipality, Eastern Cape Province, South Africa. Source: Omoruyi et al. [12].

The aim of this study was to document the plant species used exclusively for the treatment of TB by the traditional healers in selected areas of Nkonkobe Municipality, Eastern Cape Province of South Africa (Figure 1).

## 2. Methodology

### 2.1. Study Area

The study area falls within Amathole district area latitudes from 30°00 to 34°15′S and longitudes from 22°C 45′ to 30°15′E. This area consists of many villages which are generally described as rural and poor with high prevalence of TB.

An ethnobotanical survey on medicinal plants was carried out in order to obtain phytotherapeutic information on plants useful for the treatment of tuberculosis in Eastern Cape Province, South Africa. This survey was conducted in the following villages within the Nkonkobe Municipality: Hala, Ncera, Sheshegu, and Gquamashe, using a well-structured questionnaire. One hundred informants were interviewed using semistructured questionnaire in order to obtain information from the rural dwellers including traditional healers known as* Sangomas* in xhosa speaking communities. Knowledgeable village elders who use medicinal plants were also consulted to provide information on the medicinal plants and their importance in the treatment of tuberculosis. This method proved to be a very viable and an effective option of data collection. The choice to employ this particular method was heavily influenced by the literacy levels, remote locations visited, and willingness of the respondents that supplied the information needed for the study. The questionnaire was designed to elicit information on the demographic structures of the respondents, names of plants commonly used in the treatment of tuberculosis, plant parts used, the methods of preparation, and therapeutic application including details on administration and dosages. Interviews were conducted in the local language of the respondents and, with the help of an interpreter, it was translated to English. Samples of the plant material used for the management of tuberculosis were collected from the wild. Scientific identification of samples collected was done in the Department of Botany, University of Fort  Hare, Giffen Herbarium, where herbarium specimens are kept; and thereby reference to standard botanical classification and nomenclature [15, 16]. Identification was further confirmed by Professor D. S. Grierson, Department of Botany, University of Fort Hare. Plant species were grouped into their respective families along with local and common names.

### 2.2. Data Analysis

For quantitative analysis, an ethnobotanical index was used to evaluate the local importance of those species in the study area using the relative frequency of citations (RFC).

The ethnobotanical data on plant species collected for this study were elaborated and analysed. The different plant species were grouped into their respective families, with information on most cited species, plant parts used method of preparation, therapeutic application, dosage, mode of use and mode of treatment was also provided. Quantitative analysis of the data was done to know the diversity of species of plants in the study area, to verify the potential of local knowledge (importance) of the communities studied. Therefore, from the citations, the number of species, the number of respondents who gave some information, and information on the plant species were supplied. The ethnobotanical indices are found on the basic structure of the ethnobotanical information. Data analyses were followed by ethnobotanical indexes using relative frequency of citation.

Relative frequency of citation (RFC) is used to find out probability between number of people who give citation to each species and number of all respondents. The result described local importance of each species. RFC was calculated thus

(1) $$\begin{array}{c} \text{RFC}=\frac{\text{FC}}{N}, \end{array}$$

where RFC is the relative frequency of citation, FC is the number of respondents who gave citations at each species, and* N* is the number of respondents.

### 2.3. Intellectual Property Agreement Statement

The traditional healers who participated and also shared their wealth of knowledge on the information of plant usage during the ethnobotanical survey were adequately informed that this research shall not be for commercial purposes but will serve as a way of conserving indigenous knowledge as regards the traditional management of tuberculosis in Nkonkobe Municipality, Eastern Cape, South Africa. Ethical approval for the study was granted by the University of Fort Hare's Ethics Committee (UREC).

## 3. Results

In this study, 80% of the respondents were males and 20% were females (Table 1). Generally, older people (34%) were mostly engaged in the practice of using herbs for healing. This culture is still prominent in Africa. 80% of the respondents claimed to have inherited the healing knowledge from their parents and has become a norm in the family. Out of the 100 respondents, a total of 80 males (80%) and 20 females (20%) were interviewed. Most of the respondents were between the ages of 21 and 80. Only 8 respondents were between the ages of 21 and 30, 9 between 31 and 40, 12 respondents between 41 and 50, 19 were between 51 and 60, 20 respondents were between 61 and 70, and 32 respondents between 71 and 80. The majority of the traditional medicine practitioners had primary school education with 78% out of the total respondents, while 10% had secondary school education, 2% out of the total respondents had vocational education/training, and 10% had no education. This is an indication that in the study area (Nkonkobe Municipality) education within the tradomedical practitioner is still at infancy. Furthermore, the result revealed that all the respondents have experience in the treatment of TB in the villages. We are also informed that the patients are diagnosed by observing the rate of coughing or cough sputum with blood stain.

A total of 30 plant species belonging to 21 families were indicated as being used traditionally for the treatment of TB (Table 2). Rutaceae and Alliaceae have the highest proportion of species used for the treatment of TB and associated diseases. At least one of these plants was mentioned by two or more respondents to be contained in their recipes for the treatment of TB; of these plants, the most frequently mentioned were* Clausena anisata* Hook,* Haemanthus albiflos *L*, Artemisia afra* Jacq. ex Willd,* Carpobrotus edulis* (L.) Bolus*, Ptaeroxylon obliquum *Thunb, and* Tulbaghia violaceae *Harv. (Table 3).

**Figure 2:**
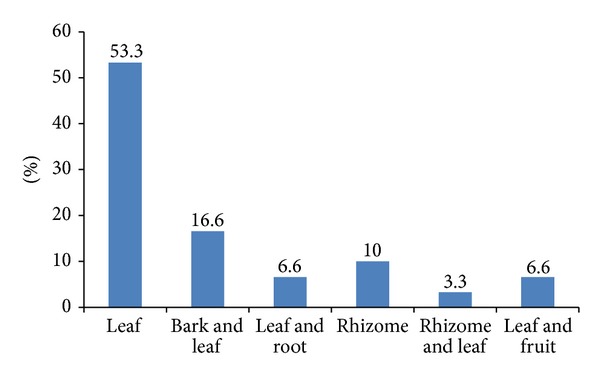
Variation in the parts of the plants used in the treatment of ailments.

The plant parts used in most of the herbal preparations include the leaves, leaves combined with bark, leaves combined with root, rhizome combined with leaves, and fruit combined with leaves. The leaves were the most frequently used (53.3%) (Figure 2) followed by the bark combined with leaves (16.6%) and rhizome (10%). The herbal medical practitioners, however, indicated that leaves are more effective than the other parts of the plants. They also claimed that it will take about 1–4 months for a TB patient to be healed during treatment. According to the healers, the healing of a patient begins with the reduction in chronic coughing and bloody sputum. An important finding from this study is that the inhabitants of the villages consult herbal practitioners because of their belief in holistic nature of treatment and the cost of treatment which is relatively cheaper than the orthodox medicines. Another finding was that the herbal medical practitioners believe that certain ailments are cured with the aid of herbal medicine and consultation with the ancestors for bewitched persons who are believed to be difficult to manage with orthodox medicine.

### 3.1. Local Importance of the Plant Species Sampled

Based on the local importance analysis of each of the plant species used for the treatment of TB in the four villages in Nkonkobe Municipality (Table 4),* Clausena anisata *was the most useful plant species (RFC = 0.1) with 10 citations (10% of the informants). It was followed by* Haemanthus albiflos *(RFC = 0.07) with 7 citations, and also* Artemisia afra *(RFC = 0.006)* Clausena anisata* was the most cited plant in this study. The recipes for the treatment of tuberculosis within the study area were made up of plant parts such as leaves, bark, fruit, and root only. These recipes were prepared as concoctions, macerations, and infusions while the mode of administration is oral (Table 5).

## 4. Discussion

### 4.1. Plant Use Based on Indigenous Knowledge

Indigenous knowledge (IK) is one of unique experiences applied to traditional knowledge that is transferred to younger generation and is still developed by rural indigenous communities in specific geographical areas. The characteristics of IK come into view and are developed in specific society; they are unique and exclusive [17].

The observation that a greater proportion of the informants were males could be attributed to the fact that male respondents were bold and courageous to talk and have rapport with the interviewer unlike their female counterparts who prefer to shy away.

Generally, older people were mostly engaged in the practice of using herbs for healing. This information on the age of the respondents implies that youths in the study areas are not fully engaged in the traditional medicine practice and this suggests a breakdown in dissemination of knowledge between the old and younger generation. This simply implies that with time this knowledge may be lost (due to the death of the old people with vast knowledge of herbal medicine) unless efforts are made to reverse the situation.

A great proportion of the plant species documented have been validated through phytochemical and pharmaceutical research; some, although, not evaluated for their efficacy are used to treat TB and opportunistic diseases associated with tuberculosis in South Africa and other parts of the world. For instance,* Artemisia afra* is used by the traditional healers in Amathole District to treat Flu and TB, and it is also used by the Zulu people [18–20]. The leaf extract of* Haemanthus albiflos* was reported to have shown significant difference against DNA viruses and all RNA viruses [21] likewise the leaf of* Carpobrotus edulis* was reported to have showed significant difference for the treatment of TB and as immune booster for HIV patients in Nkonkobe Municipality [12, 20]. Furthermore, Lall and Meyer [22] reported that individuals infected with HIV/AIDS are also susceptible to TB and often develop this disease before other manifestations become apparent.

*Cannabis sativa* was also reported, through infusion and inhalation, to be used for the treatment of TB among the Zulu people, Hutchings et al. [23]. Leaf extract of* Eucalyptus camaldulensis *inhibited the growth of* Bacillus cereus*,* Escherichia coli*,* Klebsiella pneumonia*, and* Staphylococcus aureus *[24]. Madikizela et al. [25] documented* Asparagus africanus* and* Ficus sur *which showed positive result against* Mycobacterium tuberculosis*; similarly, Ghosal et al. 1985 [26] reported that species in the* Haemanthus *genus produce several alkaloids which have therapeutic effect against coughs and dropsy asthma and as topical antiseptics. Also extracts from several species of Amaryllidaceae plants have been found to possess pronounced antibacterial and antifungal activities [27].

To the best of our knowledge* Clausena anisata, Haemanthus albiflos, Asparagus africanus, Araujia sericifera, Scabiosa albanensis*, and* Silene undulate *were recorded for the first time for the management of TB in Nkonkobe Municipality, Eastern Cape Province. This investigation plays a significant role in the medicinal plant research in the context of management of  TB and its opportunistic diseases in Eastern Cape Province. Hence, the above listed plants are widely used for treating different ailment; for instance* C. anisata* was reported to treat measles and bronchial problem in Nigeria [28]. In Tanzania, traditional healers use* Clausena anisata* against oral candidiasis and fungal infections of the skin [29].

Hutching et al. [23] reported that* C. anisata* leaf have been used for the treatment of respiratory ailments. This finding is in line with the present study which justifies the widespread use of* C. anisata* for the treatment of TB in Nkonkobe Municipality. York et al. [30] reported that the leaf extract of the plant was tested against* Cryptococcus neoformans, Klebsiella pneumonia, Moraxella catarrhalis, Mycobacterium smegmatis, *and* Staphylococcus aureus* in KwaZulu-Natal. The aqueous and methanol leaf extract of* C. anisata* was also reported in Ethiopia to have exhibited anti-antimycobacterium properties against* Mycobacterium tuberculosis* and* Mycobacterium bovis.*

## 5. Conclusion

The current study was undertaken to investigate local communities in Nkonkobe Municipality, Eastern Cape Province, as regard the treatment and management of TB. The documented medicinal plants used by the Xhosa herbalist reflect a rich ethnomedicinal knowledge in the municipality. These results strengthen the firm belief that traditional medicines are readily accessible and still play an important role in meeting the basic health care of many people in African communities.

Phytomedicinal information on the treatment of TB in this region is well established. Thirty plants belonging to 21 families were mentioned to be used for the treatment of TB and associated diseases. Other diseases treated using these plants were respiratory infections. The commonly mentioned species are* Clausena anisata*,* Haemanthus albiflos, Artemisia afra, Carpobrotus edulis, Ptaeroxylon obliquum, *and* Tulbaghia violacea.* The most frequently mentioned species was* Clausena anisata* known locally as* Iperepes*. Many studies have revealed some bioactive chemical compositions in these plants which probably justify their pharmacological properties. The following five species were recorded for the first time for the management of TB in Eastern Cape Province, South Africa:* Clausena anisata, Haemanthus albiflos, Araujia sericifera, Scabiosa albanensis, and Silene undulate.* This study has contributed to the scientific documentation of medicinal plants used for the treatment of TB. This is necessary in the rural communities to avert the erosion of traditional medicine knowledge. The larger percentage of the traditional healers is old people; therefore, this legacy needs to be conserved. Further studies are in progress on the antituberculosis assay to validate ethnopharmacology relevance of the most mentioned plants in the study area.

## Figures and Tables

**Table 1:** Demographic characteristics of the respondents in the study area.

Characteristics	Frequency	Percentage (%)
Age (years)		
Less than 20	0	0
21–30	8	8
31–40	9	9
41–50	12	12
51–60	19	19
61–70	20	20
71–80	32	32

Total	100	100

Gender		
Male	80	80
Female	20	20

Total	100	100

Sources of information		
Ancestral	80	80
Others	20	20

Total	100	100

Years of experience		
Less than 5	3	3
6–10	4	4
11–15	7	7
16–20	20	20
Above 21	66	66

Total	100	100

Experience in treating TB		
Yes	100	100
No	0	0

Total	100	100

Level of education		
Primary education	78	78
Secondary education	10	10
Adult/vocational education	2	2
No education	10	10

Total	100	100

**Table 2:** Distribution of plants family showing their percentage occurrence.

Family	Distribution	% occurrence
Alliaceae	3	10
Rutaceae	3	10
Apiaceae	2	6.6
Asteraceae	2	6.6
Lamiaceae	2	6.6
Myrtaceae	2	3.3
Rosaceae	2	6.6
Solanaceae	2	6.6
Aizoaceae	1	3.3
Amaryllidaceae	1	3.3
Apocynaceae	1	3.3
Asparagaceae	1	3.3
Cannabaceae	1	3.3
Caryophyllaceae	1	3.3
Dispaceae	1	3.3
Fabaceae	1	3.3
Hypoxidaceae	1	3.3
Moraceae	1	3.3
Rhamnaceae	1	3.3
Rubiaceae	1	3.3
Sapindaceae	1	3.3

**Table 3:** Distribution of plant species according to the ethnobotanical survey.

Species	Number of occurrences
*Clausena anisata *	10
*Haemanthus albiflos *	7
*Artemisia afra *	6
*Cannabis sativa *	5
*Carpobrotus edulis*	5
*Mentha longifolia *	5
*Ptaeroxylon obliquum *	5
*Tulbaghia violacea *	5
*Agathosma betulina *	4
*Hippobromus pauciflorus *	4
*Hypoxis argentea *	4
*Allium spp. *	3
*Capsicum frutescens *	3
*Corymbia citriodora *	3
*Daucus carota *	3
*Ficus spp. *	3
*Rosmarinus officinalis *	3
*Scabiosa albanensis *	3
*Tulbaghia acutiloba *	3
*Withania somnifera *	3
*Acacia karroo *	2
*Araujia sericifera *	2
*Bidens pilosa *	2
*Asparagus africanus*	1
*Centella coriacea*	1
*Prunus africana *	1
*Rubia petiolaris *	1
*Silene undulata *	1
*Syzygium cordatum*	1
*Ziziphus mucronata *	1

**Table 4:** Result of analysis used by RFC for the medicinal plant species. *n* = 100.

No.	Species	Basic value		Ethnobotanical index	Ranking of RFC
		FC	*N* (%)	RFC	
1	*Clausena anisata *	10	10	0.1	1
2	*Haemanthus albiflos *	7	7	0.07	2
3	*Artemisia afra *	6	6	0.06	3
4	*Cannabis sativa *	5	5	0.05	4
5	*Carpobrotus edulis*	5	5	0.05	4
6	*Mentha longifolia *	5	5	0.05	4
7	*Ptaeroxylon obliquum *	5	5	0.05	4
8	*Tulbaghia violacea *	5	5	0.05	4
9	*Agathosma betulina *	4	4	0.04	5
10	*Hippobromus pauciflorus *	4	4	0.04	5
11	*Hypoxis argentea *	4	4	0.04	5
12	*Allium spp. *	3	3	0.03	6
13	*Capsicum frutescens *	3	3	0.03	6
14	*Corymbia citriodora *	3	3	0.03	6
15	*Daucus carota *	3	3	0.03	6
16	*Ficus spp. *	3	3	0.03	6
17	*Rosmarinus officinalis *	3	3	0.03	6
18	*Scabiosa albanensis *	3	3	0.03	6
19	*Tulbaghia acutiloba *	3	3	0.03	6
20	*Withania somnifera *	3	3	0.03	6
21	*Acacia karroo *	2	2	0.02	7
22	*Araujia sericifera *	2	2	0.02	7
23	*Bidens pilosa *	2	2	0.02	7
24	*Asparagus africanus*	1	1	0.01	8
25	*Centella coriacea*	1	1	0.01	8
26	*Prunus africana *	1	1	0.01	8
27	*Rubia petiolaris *	1	1	0.01	8
28	*Silene undulata *	1	1	0.01	8
29	*Syzygium cordatum*	1	1	0.01	8
30	*Ziziphus mucronata *	1	1	0.01	8

FC: number of informant who gave citation at each species, *N* (%): the number of participants mentioning the use of the plant species as medicine for the treatment of tuberculosis in percentage of the total participants, RFC: the relative frequency of citation.

**Table 5:** Plant species used for the treatment of TB.

Botanical name	Family	Local name	Plant part used	Method of preparation	Therapeutical application	Dosage, mode of use, and duration of the treatment	Development of plant for use	Plant status
*Agathosma betulina* (Berg)	Rutaceae	Ibuchu	Leaf	3000 mL of boiled poured on 500 g tender leaves collected freshly; it was allowed to steep for 30 minutes before taken	Chronic cough	150 mL of the extract taken orally 2 × 2 for 3 weeks	Tender leaf	Fresh

*Artemisia afra* (Jacq. ex Willd)	Asteraceae	Umhlonyane	Leaf	2000 mL of boiled water was poured on 400 g tender leaves collected freshly; it was allowed to steep for 30 minutes before taken	Flu, excessive cough, tuberculosis	100 mL of the extract taken orally 2 × 1 for 2 weeks	Tender leaf	Fresh

*Acacia karroo* (Hayne)	Fabaceae	Umnga	Bark and leaf	2000 mL of hot water poured on 400 g air dried leaves, for 25 minutes, while the grounded bark can be also infused alternatively	Tuberculosis	75 mL of the infused plant substance taken orally. 1 × 1, for 1 month	Tender and mature	Air dried

*Ptaeroxylon obliquum* (Thumb)	Rutaceae	Umpafa	Leaf	The air dried leaves of the plant left in contact with the menstruum (Alcohol) for five days which was later sieve to get the extract for the treatment	Tuberculosis and Chest complaints	100 mL of the extract taken orally 3 × 1, for 3 weeks	Tender leaf	Air dried

*Tulbaghia violacea* (Harv.)	Alliaceae	Clausena anisata	Rhizome	This was extemporaneously prepared; cold water was added to 1000 g of air dried herbal substance and boiled under reduced temperature for 45 minutes	Ulceration of the lung	150 mL taken orally 3 × 1, for 3 weeks	Mature	Air dried

*Prunus africana* Hook.	Rosaceae	Umkakase	Bark and leaf	This was extemporaneously prepared; cold water was added to 1000 g of air dried bark and leaves of herbal substance, boiled under reduced temperature for 45 minutes	Whooping cough and Tuberculosis	150 mL taken orally for 3 × 1, for 3 weeks	Tender and mature	Air dried

*Clausena africana* (Hook)	Rutaceae	Ipereres	Leaf and Bark	2000 mL of hot water will be poured on 400 g air dried leaves, for 25 minutes. The grounded bark can also be infused alternatively	Tuberculosis and chest complaints	150 mL taken orally for 3 × 1, for 2 weeks	Tender leaf	Air dried

*Haemanthus albiflos* (L)	Amaryllidaceae	Istitibala	Leaf and root	The air dried leaf and bark of the plant will be soaked in alcohol for six days and will be shaken occasionally	Tuberculosis	75 mL taken orally 3 × 1, for 3 weeks	Mature and tender	Air dried

*Mentha longifolia* (L)	Lamiaceae	Inxina	Leaf	2000 mL of boiled water was poured on 400 g tender leaves collected freshly, allowed to steep for 30 minutes before taken	Ulceration of the lung	100 mL of herb taken orally 3 × 1 for 2 month	Tender leaf	Fresh

*Hypoxis argentea* (Fiscand)	Hypoxidaceae	Inongwe	Leaf	2000 mL of hot water will be poured on 400 g of fresh leaves and allowed to steep for 30 minutes	Tuberculosis	100 mL of the prepared herbs taken 3 × 1 for 3 weeks	Tender leaf	Fresh

*Ficus sur* (Forrssk)	Moraceae	Mngxam	Leaf	2000 mL of hot water poured on 400 g of fresh leaves and allowed to steep for 30 minutes	Ulceration of the lung and tuberculosis	100 mL of the prepared herb will be taken orally 3 × 1 for 1 month	Tender leaf	Fresh

*Hippobromus pauciflorus* (Radlk)	Sapindaceae	Mfazionegxolo	Leaf	2000 mL of hot water poured on 400 g of fresh leaves and allowed to steep for 30 minutes	Tuberculosis	100 mL of prepared herbs taken 3 × 1, for 2 months	Mature	Fresh

*Araujia sericifera* (Brot)	Apocynaceae	Impinda	Rhizome and leaf	2000 mL of hot water poured on 400 g of fresh leaves and matured rhizome of the plant and allowed to steep for 30 minutes	Tuberculosis	75 mL of prepared herbs taken orally 2 × 1, for 2 months	Mature	Fresh

*Allium sativum* (L)	Alliaceae	Ikoronofile	Rhizome	2000 mL of hot water poured on 400 g of fresh rhizomes and allowed to steep for 30 minutes	Bloody cough and ulceration of the lung	50 mL of the prepared herbs taken 2 × 1, for 3 weeks	Mature	Fresh

*Rosmarinus officinalis* (L)	Lamiaceae		Leaf	2000 mL of hot water will be poured on 400 g of matured leaves and allowed to steep for 30 minutes.	Tuberculosis	150 mL of the prepared herbs taken 3 × 1 for 3 weeks	Mature	Fresh

*Cannabis sativa* (L)	Cannabaceae	Umya	Leaf	2000 mL of hot water poured on 400 g of matured leaves and allowed to steep for 30 minutes	Tuberculosis	150 mL of prepared herbs will be taken orally 3 × 1 for 3 weeks	Mature	Fresh

*Daucus carota* (L)	Apiaceae		Leaf and fruit	2000 mL of hot water will be poured on 400 g of matured leaves and allowed to steep for 30 minutes. Or the leaves and the fruit can be soaked with alcohol for seven days	Tuberculosis	75 mL of the prepared herbs taken orally 3 × 1, for 1 month	Mature and tender	Air dried

*Bidens pilosa* (L)	Asteraceae	Imbikicane	Leaf and bark	This was extemporaneously prepared; cold water was added to 1000 g of air dried bark and leaves of herbal substance and boiled under reduced temperature for 45 minutes	Tuberculosis	75 mL of prepared herbs taken orally 3 × 1, for 3 weeks	Mature and tender	Air dried

*Corymbia citriodora* (L)	Myrtaceae		Leaf	2000 mL of hot water will be poured on 400 g of tender leaves and allowed to steep for 30 minutes	Tuberculosis	75 mL of prepared herbs taken orally 3 × 1, for 3 weeks	Tender leaf	Fresh

*Ziziphus mucronata* (Willd subsp.)	Rhamnaceae		Leaf	2000 mL of hot water poured on 400 g of tender leaves and allowed to steep for 30 minutes	Tuberculosis	75 mL of the prepared herbs taken 3 × 1, for 1 month	Tender leaf	Fresh

*Capsicum frutescens* (L)	Solanaceae	Ikhanakhana	Fruit and leaf	This was extemporaneously prepared; cold water was added to 1000 g of air dried fruit and leaves of herbal substance and boiled under reduced temperature for 35 minutes	Tuberculosis	50 mL of the prepared herbs taken orally 2 × 1, for 3 weeks	Mature	Air dried

*Withania somnifera* (L) Dunal	Solanaceae	Ubuvimba	Leaf	2000 mL of hot water poured on 400 g of tender leaves and allowed to steep for 30 minutes	Tuberculosis	150 mL of prepared herbs taken orally 3 × 1 for 3 weeks	Tender leaf	Fresh

*Silene undulata* (L)	Caryophyllaceae	Isilawu	Leaf	2000 mL of hot water poured on 400 g of tender leaves and allowed to steep for 30 minutes	Tuberculosis	75 mL of the prepared herbs taken orally 3 × 1, for 1 month	Tender leaf	Fresh

*Scabiosa albanensis* (L)	Dipsacaceae	Umsilawu	Leaf and root	The root and leaves of the plant left in contact with the menstruum (alcohol) for five days which was later sieved to get the extract for the treatment	Tuberculosis	50 mL of the extract taken orally 3 × 1, for 1 month	Mature and Tender	Fresh

*Rubia petiolaris* (DC)	Rubiaceae	Impendulo	Leaf	2000 mL of hot water poured on 400 g of tender leaves and allowed to steep for 30 minutes	Tuberculosis	150 mL of the prepared herbs taken 3 × 1 for 3 weeks	Tender leaf	Fresh

*Tulbaghia acutiloba* (Harv.)	Alliaceae	Isivumbampunzi	Rhizome	2000 mL of hot water poured on 400 g of air dried rhizomes and allowed to steep for 30 minutes	Tuberculosis	150 mL of the prepared herbs taken 3 × 1 for 3 weeks	Mature	Air dried

*Asparagus africanus* (Lam)	Asparagaceae	Ikhubalo	Leaf	Infusion 2000 mL of hot water poured on 400 g of tender leaves and allowed to steep for 30 minutes	Tuberculosis	150 mL of the prepared herbs taken orally 3 × 1 for 3 weeks	Tender leaf	Fresh

*Centella coriacea* (L)	Apiaceae	Unongotyozana	Leaf	2000 mL of hot water poured on 400 g of fresh leaves and allowed to steep for 30 minutes	Tuberculosis	100 mL of the prepared herbs taken orally 3 × 1 for 3 weeks	Tender leaf	Fresh

*Carpobrotus edulis* (L) Bolus	Aizoaceae	Unomatyumtyum	Leaf	2000 mL of hot water will be poured on 400 g of fresh leaves and allowed to steep for 30 minutes	Tuberculosis	100 mL of the herbs taken orally 3 × 1 for 3 weeks	Tender leaf	Fresh

*Eucalyptus camadulensis *	Myrtaceae	Gumtriya	Leaf and bark	2000 mL of hot water poured on 400 g of fresh leaves and barks and allowed to steep for 45 minutes	Tuberculosis/constant coughing	100 mL taken orally 3 × 1 for 3 weeks	Mature and tender	Fresh

Key: 3 × 1: thrice daily; 2 × 1: twice daily; 1 × 1: once daily.
